# Developing and testing a cognitive bolt-on for the EQ-5D-Y (Youth)

**DOI:** 10.1007/s11136-021-02899-x

**Published:** 2021-06-10

**Authors:** Kristina Ludwig, Bastian Surmann, Eva Räcker, Wolfgang Greiner

**Affiliations:** Health Economics and Health Care Management, School of Public Health, Bielefeld University, Universitätsstraße 25, 33615 Bielefeld, Germany

**Keywords:** EQ-5D-Y, Bolt-on, Cognitive dimension, Health-related quality of life, Adolescents, Children

## Abstract

**Purpose:**

The aim of this study was to develop and test a cognitive dimension as a bolt-on for the German version of the EQ-5D-Y (Youth).

**Methods:**

A literature review and six focus groups with children and adolescents were used to develop the cognitive dimension for the EQ-5D-Y. In a two-phase pretest, the acceptability, feasibility and performance of this dimension were assessed (phase 1: qualitative face-to-face interviews, phase 2: standard pretest in a clinical setting). In total, 280 children and adolescents participated in this study.

**Results:**

School performance, concentration, memory and learning ability represent the most important components of cognitive functioning in children and adolescents. Hence, those components were incorporated into the cognitive dimension of the EQ-5D-Y by adding four items. For children and adolescents living with a rheumatic disorder or type 1 diabetes mellitus, the EQ-5D-Y plus a cognitive bolt-on demonstrated good acceptability, feasibility and performance. The cognitive items improved the explanatory power for the EQ visual analogue scale (EQ-VAS). Factor analysis has shown that a reduction of the cognitive bolt-on into one or two item(s) is justifiable.

**Conclusion:**

By enhancing the EQ-5D-Y with a cognitive bolt-on, we developed an instrument that incorporates current findings on Health-Related Quality of Life (HRQoL) and is suitable for the target population. Empirical results of this study show that cognitive functioning is an important part of HRQoL assessment in children and adolescents. The inclusion of a cognitive dimension in the EQ-5D-Y improves the HRQoL measurement.

**Supplementary Information:**

The online version contains supplementary material available at 10.1007/s11136-021-02899-x.

## Background

Due to the increasing importance of assessing Health-Related Quality of Life (HRQoL) in children and adolescents in clinical practice and research, demand for suitable measuring instruments is growing [1–4]. To appropriately measure HRQoL, these instruments should include all dimensions relevant to their target group [4, 5]. In this context, the measurement of cognitive functioning is neglected to some extent, e.g. in the EQ-5D-Y (Youth) developed by the EuroQol Group. The EQ-5D-Y is based on the adult three-level version of the EQ-5D (EQ-5D-3L) and similarly includes a descriptive system and the EQ visual analogue scale (EQ-VAS). The descriptive system comprises five HRQoL dimensions (mobility, looking after myself, doing usual activities, having pain or discomfort and feeling worried, sad or unhappy), assessed by three levels of severity [no (level 1), some (level 2), a lot of problems (level 3)] [6, 7]. The EQ-5D-Y was simply adapted from the adult measure by experts rather than engaging with children and adolescents to understand how they conceptualize their own HRQoL. As this might have resulted in the exclusion of HRQoL aspects relevant to children and adolescents [6], research is needed to examine how the addition of dimensions may improve the EQ-5D-Y’s performance.

Children and adolescents are under pressure to perform well considering the increasingly achievement-oriented society (e.g. in school) [8, 9]. Thus, there is growing awareness and discussion of developmental disorders of scholastic skills (e.g. dyslexia) and related conditions such as Attention Deficit Disorder in Germany [10]. As HRQoL encompasses cognitive functioning and role functioning [11], these disorders should have a large impact on HRQoL. Considering the pressure to perform, chronic health conditions and acute illness may directly or indirectly affect cognitive function and consequently the HRQoL of the child or adolescent [12–14]. The EQ-5D-Y does not contain an explicit cognitive dimension although “doing usual activities” may partly cover cognitive aspects by including the example “going to school”. However, it is unclear whether children consider their cognitive function while answering this item. Krabbe et al. (1999) examined the effect of adding a cognitive dimension to the EQ-5D-3L in adults [15]. This study showed the importance of testing the inclusion of a cognitive dimension. Supported by theoretical and practical arguments, the authors recommended the inclusion of a cognition attribute within the EuroQol classification.

Considering the impact of cognitive function on HRQoL in childhood and adolescence, its absence in the EQ-5D-Y is a potential limitation of the instrument. Therefore, the objectives of this study are to

1. develop and test a pilot cognitive dimension as a bolt-on for the German version of the EQ-5D-Y,
2. test the acceptability, feasibility and performance of the bolt-on cognitive dimension (EQ-5D-Y plus bolt-on).

**Fig. 1:**
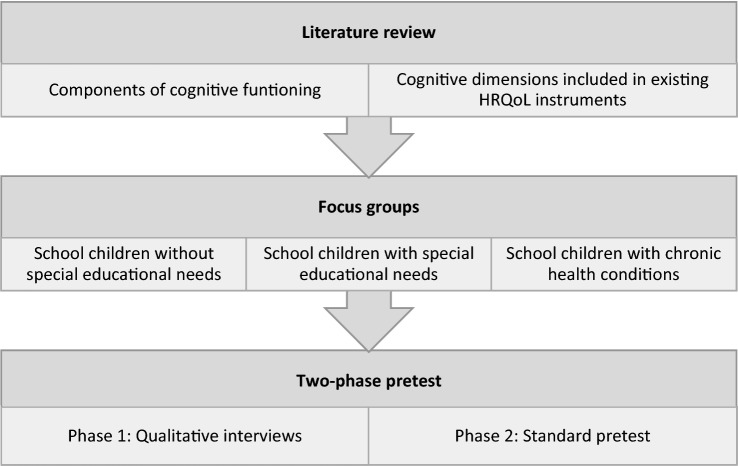
Methodological procedure

The study was conducted in Germany between 2010 and 2019. Two literature reviews and focus groups were used to develop the bolt-on. In a subsequent two-phase pretest, the acceptability, feasibility and functioning of the bolt-on was assessed through qualitative face-to-face interviews and a standard pretest of the measure in a clinical setting (Fig. 1, detailed methodological procedure in Online Appendix 1).

## Developing a cognitive bolt-on for the EQ-5D-Y

### Methods

#### Procedure

Two sequential non-systematic literature reviews were conducted to identify, (1) components of cognitive functioning, and (2) items and dimensions encompassing cognitive functioning included in existing HRQoL instruments for children and adolescents (Fig. 1). The selection of instruments was based on two existing literature reviews of HRQoL instruments [4, 16] and a manual literature search. These results were included in the topic guide for the focus groups.

Focus groups with children and adolescents (aged 8 to 11, and 12 to 15) were conducted to investigate the understanding and importance of components of cognitive functioning, and to identify items for the bolt-on. Each focus group included discussion around 1) their daily activities, 2) the impact of (their) disease on daily activities, 3) components of cognitive functioning and finally 4) the components of cognitive functioning were weighted using the nominal group technique (NGT) [17, 18].

Items were selected based on results of the literature reviews and focus groups.

#### Data analysis

The focus groups were recorded and transcribed, then analysed using qualitative content analysis according to Mayring [19]. A pool of possible items for the bolt-on was derived from the participants’ statements. Items were reduced and selected by the research team using the card sorting procedure [20]. The analysis of the focus groups was stratified by age group to investigate the need for different age versions.

The components of cognitive functioning and the respective items were then selected for the pilot bolt-on with consideration of the results from both literature reviews, the qualitative analysis and NGT of the focus groups. The items were developed with consideration to the relevance to the target group by incorporating components of cognition with examples taken from the children and adolescent’s explanation/understanding of the items. The wording, structure and design of the bolt-on items followed the EQ-5D-Y.

### Results

#### Literature reviews

Cognitive skills include ten components of cognitive functioning: school performance, concentration, memory, learning ability, rationality, speech, social adaptability, problem solving, orientation and emotions [12, 21, 22]. The most frequently used HRQoL instruments Child Health Questionnaire (CHQ) [23]; Child Health and Illness Profile (CHIP) [24]; How are you? (HAY) [25], KIDSCREEN [26, 27]; KINDL-R [28]; Paediatric Quality of Life Inventory (PedsQL) [29, 30] and TNO/AZL Child Quality of Life (TACQOL) [31] often include components of school performance and concentration.

#### Focus groups

Thirty-seven children and adolescents participated in six focus groups in April and May 2010, until information saturation was reached. Both age groups were evenly represented (Table 1). Participants with special educational needs had delayed cognitive development and/or a (partial) disruption of cognitive abilities. Children with a chronic condition had been diagnosed with asthma (*n* = 5), diabetes (*n* = 4), cystic fibrosis (*n* = 1) or cancer (*n* = 1).

**Table 1:** Demographics of participants

	Focus groups (*n* = 37)	Phase 1 Qualitative pretest (*n* = 20)	Phase 2 Standard pretest (*n* = 223)
Sex, *n* (%)			
Male	20 (54)	11 (55)	93 (42)
Female	17 (46)	9 (45)	127 (58)
Age groups, years, *n* (%)			
8–11	18 (49)	9 (45)	91 (41)
12–15	19 (51)	11 (55)	132 (59)
Educational need/ health status, *n* (%)			
School children	15 (41)	7 (35)	–
Special educational need	11 (30)	7 (35)	–
(Chronic) health condition	11 (30)	6 (30)	–
Health condition, *n* (%)			
Rheumatic disorder	–	–	83 (37)
Type I diabetes	–	–	140 (63)

Content analysis of the focus groups suggested that school performance, concentration, memory, learning ability and rationality represent the components of cognitive functioning that were best understood and representative for the included children and adolescents. According to the participants, these five components may be affected by illness and subsequently influence HRQoL. Participants considered these components a precondition to not having impairments (e.g. being healthy), rather than pre-existing or self-evident. All participants reported having problems with components of school performance, concentration, memory, learning ability and rationality in previous periods of illness. Participants judged these experiences negatively. The ranking of components showed that concentration, memory and learning ability are the most relevant cognitive components. Comparison of age groups showed that school performance and social adaptability are more relevant in the younger group.

#### Selection of components and items

The results of the literature reviews, the qualitative analysis and NGT results of the focus groups indicate that school performance, concentration, memory and learning ability represent the most important components of cognitive functioning (qualitative ranking in Table 2). Hence, these informed the development of the bolt-on for the EQ-5D-Y. Considering the results of the focus groups, an additional item of “cognitive abilities” was retained ensuring a general cognitive item. Social adaptability was relevant to the younger age group, but was not included in the bolt-on as content analysis showed that participants did not consider social adaptability as their own cognitive achievement but rather a precondition for other components.

**Table 2:**
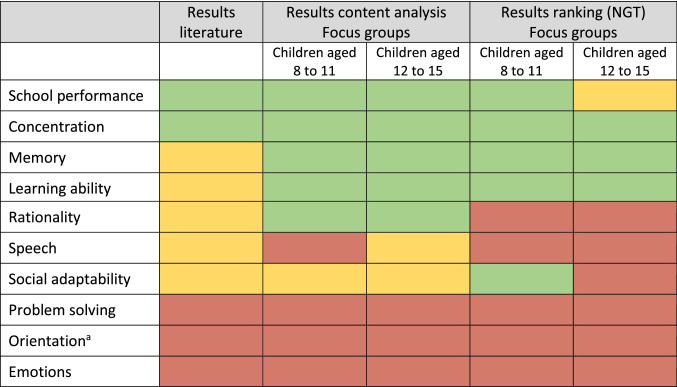
Assessment of components of cognitive functioning

Resulting recommendations: inclusion in cognitive dimension (green), possible inclusion in cognitive dimension (yellow), no inclusion in cognitive dimension (red)

^a^Awareness of place, time, person and situation

The developed bolt-on includes four items: a general item of “cognitive abilities” and specific components of concentration, memory (“remembering”) and school performance. The results of the focus groups suggested that learning ability was an application or example of the aforementioned components. Thus, learning ability was included in these three items as example. All bolt-on items include an explanation and/or examples in parenthesis to cover all relevant items identified in the item selection process. The selected bolt-on items were formatted in the EQ-5D style (i.e. wording of items, explanation in parentheses and response level).

## Pretesting of the developed EQ-5D-Y plus bolt-on in two phases

### Phase 1: Qualitative interviews

#### Methods

Phase 1 of the pretest assessed the acceptability and feasibility, in terms of comprehensibility, of the selected bolt-on items. Qualitative face-to-face interviews using cognitive techniques were conducted [32]. The interviews consisted of two parts: completing the EQ-5D-Y plus bolt-on, and semi-structured individual interviews, in which the participants were asked to judge the importance or redundancy of the cognitive items.

#### Data analysis

The participants’ responses and questions of clarification during the completion of the questionnaire were noted. The semi-structured interviews were recorded and transcribed for content analysis according to Mayring [19]. Descriptive analyses were used to examine the frequencies of reported problems for the bolt-on items. Statistical analyses were performed in R [33]. Based on the results of the qualitative pretest, the bolt-on was adapted and finalized for further testing in phase 2.

#### Results

Twenty children and adolescents participated in phase 1 of pretesting in July 2010. All children in the disease group had cancer and were receiving therapy, sample characteristics are shown in Table 1.

**Fig. 2:**
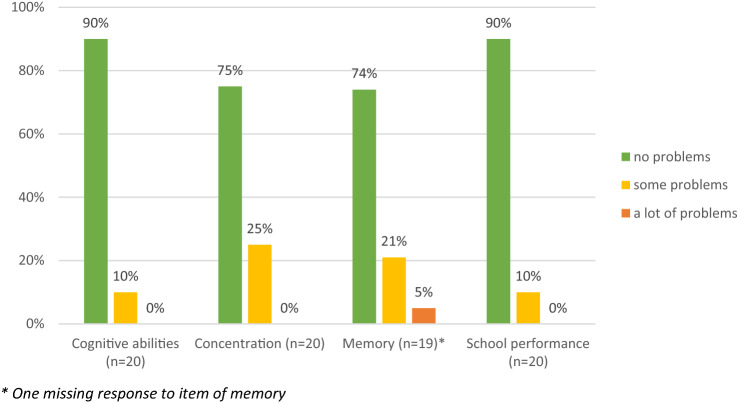
Results of phase 1 pretest—frequencies of reported problems in pilot cognitive bolt-on (in %)

The bolt-on was well accepted and considered relevant by children and adolescents. All participants completed the EQ-5D-Y plus bolt-on without assistance or clarification from the interviewer. Younger children needed more time compared to the older group (mean duration: 7.5 versus 3.5 min). The use of the EQ-5D-Y plus bolt-on revealed impairment of cognitive functioning in 10–26% of participants (Fig. 2).

**Fig. 3:**
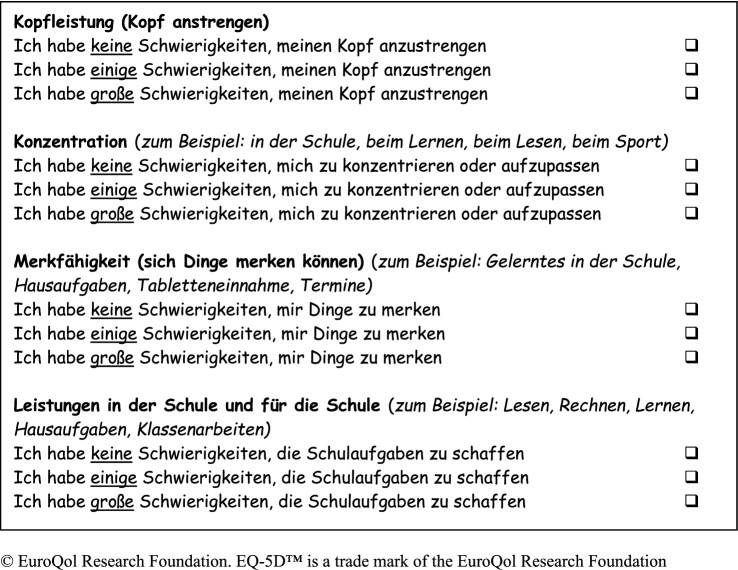
Developed cognitive bolt-on for the German version of the EQ-5D-Y*

Content analysis of the semi-structured cognitive interviews confirmed that participants had no problems completing the questionnaire. Five participants reported problems with the phrasing and/or word choice in the headings for cognitive abilities and memory. As all participants fully understood the explanations of the headings and the examples in the questionnaire (i.e. “thinking” and “remembering”), no changes to wording was necessary. Results showed that the new bolt-on dimension was clear, easily understood and free of misinterpretation. Thus, no items were deleted, merged or added. However, “appointments” was added as example to the memory item, as this was suggested by 25% of participants (*n* = 4). The developed bolt-on for EQ-5D-Y is shown in Figs. 3 (developed German version) and 4 (non-validated English translation).

**Fig. 4:**
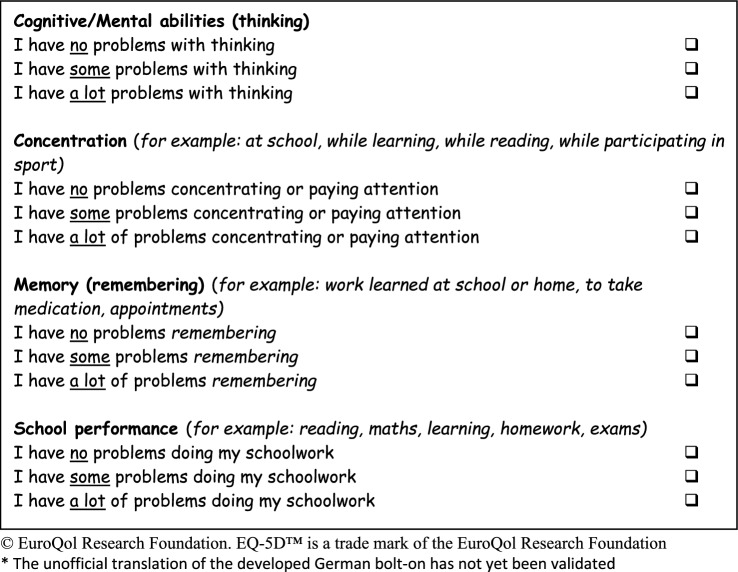
English translation of the cognitive bolt-on for the EQ-5D-Y*

### Phase 2: Standard pretest

#### Methods

In the second pretest phase, a quantitative survey was conducted in a paediatric outpatient clinic in Germany to test the acceptability, feasibility and performance of the EQ-5D-Y plus bolt-on. Children aged 8 to 15 living with type 1 diabetes mellitus (T1D) or a rheumatic disorder (RD) were enrolled. The selection of disease groups was according to the prevalence in children and adolescents and for the associated impact on cognitive function.

**Fig. 5:**
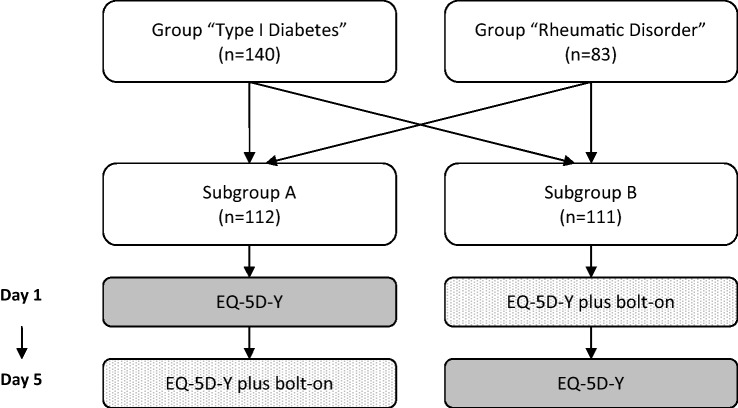
Phase 2 standard pretest—classification of study sample and procedure of questioning

Each participant self-completed the EQ-5D-Y and EQ-5D-Y plus bolt-on at two different time points: day 1 (after a medical check-up) and day 5 (at home). Participants’ HRQoL was additionally evaluated with the KIDSCREEN-27 [34]. The order of completion was randomized to reduce bias (Fig. 5).

This phase explored the following research questions:

1. Does the addition of a cognitive bolt-on improve the explanatory power of the questionnaire compared to the standard EQ-5D-Y?
2. Does the benefit of measuring HRQoL with an additional cognitive bolt-on differ between children and adolescents living with RD or T1D?

#### Data analysis

Descriptive analyses were used to examine the sample characteristics and the responses to the EQ-5D-Y and EQ-5D-Y plus bolt-on. The feasibility and acceptability of EQ-5D-Y plus bolt-on was assessed by missing values and ceiling effects. Differences between questionnaire versions and the disease groups were identified by t-test or Mann–Whitney-U-test.

The contribution of the bolt-on items to the self-rated overall health status measured by the EQ-VAS was explored through the increment of explanatory power between two linear regression models. The first regression model included items of the EQ-5D-Y and the second items of the EQ-5D-Y plus bolt-on as coefficients. The explanatory power of the two models was measured by their respective coefficients of determination (*R*^2^, ${R}_{adj}^{2}$) and Akaike’s information criterion (AIC).

The Shannon Index (H′) and the Shannon Evenness Index (J′) were used to evaluate the discriminatory power of the bolt-on in terms of absolute and relative informativity [35, 36].

To assess the external validity of the bolt-on, Spearman rank correlations were calculated between the bolt-on items and the cognitive items from the validated KIDSCREEN-27 [7]. Internal consistency of the bolt-on items was measured by Cronbach’s α.

Factor analysis was used to test whether the items of the bolt-on could be reduced to one or two latent factors. Finally, linear regression models were fitted, in which the factors resulting from the factor analysis were included as regressors. *R*^2^, ${R}_{adj}^{2}$ and AIC of the respective models were compared to determine the items that yield the best model fit and accounted for the highest explanatory power.

All statistical analyses were performed in R version 3.6.1 [33]. The level of significance was set at *p* < 0.05.

#### Results

A total of 223 respondents with a mean age of 11.94 years participated in the standard pretest between January 2015 and December 2016. Sixty-three percent of participants were living with T1D and 37% with RD (Table 1).

The EQ-5D-Y plus bolt-on proved feasible, as 94.2% of the participants completed all descriptive items. Eighteen participants had a range of 1–6 missing values. The proportions of missing values per item were low in both questionnaire versions. The items with the fewest missing values were “mobility” and “doing usual activities” (0.4% each) in the EQ-5D-Y and similarly “mobility” and “looking after myself” (0.4% each) in the EQ-5D-Y plus bolt-on. The items with the highest missing values were “having pain or discomfort” (2.2%) and “memory” (2.2%) on the EQ-5D-Y and EQ-5D-Y plus bolt-on, respectively (Table 3). The observed ceiling effect in the EQ-5D-Y items (83.3% reported “no problems”) was higher than the bolt-on items (76.7%). The EQ-5D-Y plus bolt-on showed problems with cognitive functioning in 16.4% (“cognitive abilities”) to 31.4% (“concentration”) of participants. There were no statistically significant differences in reported problems in the EQ-5D-Y dimensions nor the EQ-VAS between questionnaires.

**Table 3:** Frequencies of reported problems and missing values for EQ-5D-Y and EQ-5D-Y plus bolt-on

EQ-5D-Y dimensions	Questionnaire version				
	EQ-5D-Y		EQ-5D-Y Bolt-On		
	(*n* = 223)		(*n* = 223)		
	*n*	%	*n*	%	
*Mobility*					p = 0.6120
No problems	197	88.3	200	89.7	
Some problems	22	9.9	22	9.9	
A lot of problems	3	1.3	0	0.0	
Missing	1	0.4	1	0.4	
*Looking after myself*					p = 0.6255
No problems	214	96.0	213	95.5	
Some problems	6	2.7	9	4.0	
A lot of problems	1	0.4	0	0.0	
Missing	2	0.9	1	0.4	
*Doing usual activities*					p = 0.5710
No problems	192	86.1	187	83.9	
Some problems	26	11.7	29	13.0	
A lot of problems	4	1.8	5	2.2	
Missing	1	0.4	2	0.9	
*Having pain or discomfort*					p = 0.7419
No problems	150	67.3	149	66.8	
Some problems	62	27.8	65	29.1	
A lot of problems	6	2.7	7	3.1	
Missing	5	2.2	2	0.9	
*Feeling worried, sad or unhappy*					p = 0.2600
Not	160	71.7	173	77.6	
A bit	57	25.6	35	15.7	
Very	4	1.8	13	5.8	
Missing	2	0.9	2	0.9	
*Cognitive abilities*					
No problems	Not included		184	82.5	–
Some problems			32	14.3	
A lot of problems			4	1.8	
Missing			3	1.3	
*Concentration*					
No problems	Not included		151	67.7	–
Some problems			62	27.8	
A lot of problems			7	3.1	
Missing			3	1.3	
*Memory*					
No problems	Not included		170	76.2	–
Some problems			43	19.3	
A lot of problems			5	2.2	
Missing			5	2.2	
*School performance*					
No problems	Not included		169	75.8	–
Some problems			47	21.1	
A lot of problems			4	1.8	
Missing			3	1.3	
*EQ-VAS*					p = 0.7507
Mean	82.4		82.69		
Standard deviation	18.69		18.02		
Missing	3	1.3	2	0.9	

Participants living with RD reported significantly more problems in the dimensions “mobility”, “doing usual activities” and “having pain or discomfort” than participants living with T1D (Table 4). For all other dimensions, including the bolt-on items, there were no significant differences between the disease groups.

**Table 4:** Frequencies of reported problems and missing values in the EQ-5D-Y plus bolt-on by disease group

EQ-5D-Y dimension	Disease group				
	Rheumatic Disorder		Type 1 Diabetes		
	*n*	%	*n*	%	
*Mobility*					***p < 0.05***
No problems	64	77.1	136	97.1	
Some problems	18	21.7	4	2.9	
A lot of problems	0	0.0	0	0.0	
Missing	1	1.2	0	0.0	
*Looking after myself*					p = 0.8220
No problems	79	95.2	134	95.7	
Some problems	3	3.6	6	4.3	
A lot of problems	0	0.0	0	0.0	
Missing	1	1.2	0	0.0	
*Doing usual activities*					p = 0.0317
No problems	64	77.1	123	87.9	
Some problems	14	16.9	15	10.7	
A lot of problems	4	4.8	1	0.7	
Missing	1	1.2	1	0.7	
*Having pain or discomfort*					p < 0.05
No problems	40	48.2	109	77.9	
Some problems	39	47.0	26	18.6	
A lot of problems	3	3.6	4	2.9	
Missing	1	1.2	1	0.7	
*Feeling worried, sad or unhappy*					p = 0.3564
Not	61	73.5	112	80.0	
A bit	13	15.7	22	15.7	
Very	7	8.4	6	4.3	
Missing	2	2.4	0	0.0	
*Cognitive abilities*					p = 0.9525
No problems	69	83.1	115	82.1	
Some problems	14	16.9	18	12.9	
A lot of problems	0	0.0	4	2.9	
Missing	0	0.0	3	2.1	
*Concentration*					p = 0.6856
No problems	55	66.3	96	68.6	
Some problems	24	28.9	38	27.1	
A lot of problems	3	3.6	4	2.9	
Missing	1	1.2	2	1.4	
*Memory*					p = 0.5544
No problems	65	78.3	105	75.0	
Some problems	14	16.9	29	20.7	
A lot of problems	2	2.4	3	2.1	
Missing	2	2.4	3	2.1	
*School performance*					p = 0.2806
No problems	60	72.3	109	77.9	
Some problems	19	22.9	28	20.0	
A lot of problems	3	3.6	1	0.7	
Missing	1	1.2	2	1.4	
*EQ-VAS*					p = 0.6197
Mean	81.88		83.17		
Standard deviation	19.58		17.09		
Missing	1		1		

*p* < 0.05 is shown in bold

Adding the bolt-on to the EQ-5D-Y increases the explanatory power (i.e. the percentage of variance of the EQ-VAS that is explained by the regression coefficients) of a linear regression model with the EQ-VAS as the dependent variable from *R*^2^ = 0.35 to *R*^2^ = 0.48 (${R}_{adj}^{2}$ improves from 0.31 to 0.43) (Table 5). This improvement in explanatory power could be observed in both disease groups:*R*^2^ (${R}_{adj}^{2}$) improves by 82% (91%) for T1D and 23% (16%) for RD. Some of the coefficients of the bolt-on, however, seem counter-intuitive, as a lot of problems with “concentration” and “memory” have a positive effect on EQ-VAS (indicating an improvement in general health).

**Table 5:** Regression analysis of EQ-VAS

	Visual Analogue Scale (EQ-VAS)	
	EQ-5D-Y	EQ-5D-Y plus bolt-on
Mobility: some problems	1.15	0.06
Mobility: a lot of problems	8.79	
Looking after myself: some problems	− 7.90	− 9.80
Looking after myself: a lot of problems	− 32.94	
Doing usual activities: some problems	− 13.43*	− 10.10*
Doing usual activities: a lot of problems	− 22.69	− 31.55*
Having pain or discomfort: some problems	− 8.59*	− 12.38*
Having pain or discomfort: a lot of problems	− 2.37	− 27.94*
Feeling worried, sad or unhappy: a bit	7.97*	− 2.50
Feeling worried, sad or unhappy: very	− 27.80*	− 14.39*
Cognitive abilities: some problems		− 5.67
Cognitive abilities: a lot of problems		− 18.14
Concentration: some problems		− 4.55
Concentration: a lot of problems		18.55*
Memory: some problems		− 2.91
Memory: a lot of problems		16.18*
School performance: some problems		3.52
School performance: a lot of problems		− 1.07
Diagnosis (rheumatic disorder)	4.69	4.05
Age	− 0.75	− 1.41*
Constant	88.61*	108.78*
*n*	213	205
*R*^2^	0.35	0.48
Adjusted *R*^2^	0.31	0.43
F Statistic	8.84* (df = 12;200)	9.45* (df = 18;186)

*p < .05

The overall discriminatory power of the EQ-5D-Y plus bolt-on is H’ = 4.41 and the relative discriminatory power is J’ = 0.31 (Table 6). The bolt-on items have a higher discriminatory power than the EQ-5D-Y items, as their relative informativity (J’ = 0.43) is higher than that of the standard items (J’ = 0.34).

**Table 6:** Shannon index (H’) and Shannon Evenness index (J’) for the items of the EQ-5D-Y plus bolt-on

	H′	J′
Mobility	0.47	0.29
Looking after myself	0.24	0.15
Doing usual activities	0.71	0.45
Having pain or discomfort	1.06	0.67
Feeling worried, sad or unhappy	0.94	0.59
Cognitive abilities	0.73	0.46
Concentration	1.05	0.66
Memory	0.87	0.55
School performance	0.87	0.55
Standard EQ-5D-Y	2.73	0.34
Bolt-on	2.75	0.43
EQ-5D-Y plus bolt-on	4.41	0.31

The bolt-on items concentration and school performance show medium-to-strong correlations to similar items on the KIDSCREEN-27, indicating convergent validity of the bolt-on: “Concentration” (bolt-on) & “Have you been able to pay attention?” (KIDSCREEN-27): ρ = 0.54. “School performance” (bolt-on) & “Have you got on well at school?” (KIDSCREEN-27): ρ = 0.40 $.$ Compared to Ravens-Sieberer et al. (2010) [7], who reported the correlations between the EQ-5D-Y and KIDSCREEN-27 items, the bolt-on items showed relatively high correlations.

Cronbach’s α for all four bolt-on items was α = 0.76, indicating an acceptable internal validity [37]. Dropping the item “memory” from the calculation results in α = 0.77, improving the original value by 0.01. Omitting any other item resulted in a reduction of $\alpha$ and would thus lower the internal validity.

**Fig. 6:**
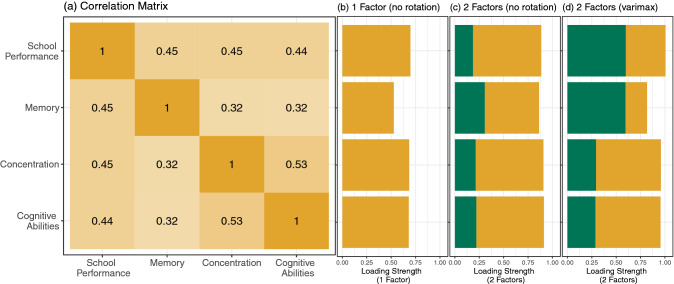
Correlation matrix and factor loadings of bolt-on items

Figure 6a shows correlation coefficients (*r*) between the bolt-on items, ranging from *r* = 0.32 to *r* = 0.53, indicating considerable correlation. Factor analysis for one factor yields factor loadings between 0.53 and 0.70 for the four items (Fig. 6b). Factor analysis with two factors, however, shows that a solution for either one dimension or for two dimensions seems to be reasonable, depending on the rotation method (Figs. 6cd). The factor loadings derived from factor analysis with varimax-rotation indicate that the items “school performance” and “memory” load onto one factor and “concentration” and “cognitive abilities” load onto a second factor.

Table 7 presents the comparison of regression models for one or two bolt-on items. When selecting one item, “memory” provides the best performance explaining the variance of the EQ-VAS (*R*^2^ = 0.42; ${R}_{adj}^{2}$=0.38) and the best model fit (AIC = 1719.4). When selecting two additional items, the best performance and model fit is achieved with “concentration” and “memory” (*R*^2^ = 0.44; ${R}_{adj}^{2}$=0.40 AIC = 1700.0). If “memory” is the only item included, *R*^2^ increases by 5%, and if “memory” and “concentration” are added, *R*^2^ increases by 11%.

**Table 7:** Regression analyses for one or two bolt-on items

		One additional item for the bolt-on				Two additional items for the bolt-on					
	EQ-5D-Y	CA	CO	ME	SP	CA + CO	CA + ME	CA + SP	CO + ME	CO + SP	ME + SP
${R}^{2}$	0.3962	0.4075	0.4154	**0.4159**	0.4019	0.4288	0.4276	0.4181	**0.4386**	0.4283	0.4238
${R}_{adj}^{2}$	0.3663	0.3716	0.3798	**0.3802**	0.3655	0.3876	0.3861	0.3761	**0.3977**	0.3870	0.3818
AIC	1752.1	1736.9	1726.0	**1719.4**	1728.0	1718.0	1712.1	1719.1	**1700.0**	1714.9	1702.6

Bold values are the lowest value of R², R²_adj or AIC

*CA* cognitive abilities, *CO* concentration, *ME* memory, *SP* school performance

## Discussion

This article presents the development and testing of a cognitive dimension as a bolt-on to the EQ-5D-Y. It provides evidence that cognitive functioning is relevant to HRQoL of children and adolescents. The absence of a cognitive dimension in the EQ-5D-Y could be related to the fact that the instrument was not specifically developed with and for the target group. Participants in both the development and testing phase of this study stated the importance of cognitive functioning and reported impairments in that dimension. The study showed that cognitive functioning has an important impact on HRQoL for children and adolescents living with both RD and T1D. In phase 2 standard pretest, only outpatients living with T1D or RD were included. Hospitalized children with more acute or severe health problems may have reported a higher level of impairments for cognition. Furthermore, other neurological or oncological diseases (e.g. meningitis, brain cancer) may have a greater impact on cognitive functioning than the selected diseases.

The EQ-5D-Y does not include a cognitive dimension even though it is relevant to children and adolescents’ HRQoL in general if one considers the influence of the increasingly achievement-oriented society [4, 13]. Furthermore, the review of dimensions and items included in frequently used generic HRQoL instruments showed that the majority include items on cognition. The number of items ranges from one (KINDL [28]) to twelve (HAY [25]), while the number of cognitive components ranges from one and four. In comparison, the EQ-5D-Y plus bolt-on includes four components of cognitive functioning and can thus be included in the higher end of the range. Similarly, to the existing instruments reviewed, the bolt-on cognitive dimension developed considers the most frequently included components of concentration and school performance. Furthermore, the EQ-5D-Y plus bolt-on is still one of the instruments reviewed with the least number of items. Following the results of the two phases of pretesting, the length of the extended questionnaire is still acceptable and will not overburden children or adolescents [5, 38]. In contrast to many the instruments reviewed, the bolt-on is not limited to items relevant to a school setting but includes a general cognitive functioning item. Other instruments that include a general item are CHQ [29] and TACQOL [31]. The addition of a cognitive bolt-on to the EQ-5D-Y gives it similar structure to the PedsQL with a generic core questionnaire and an additional Cognitive Functioning Scale [29].

The results of phase 2 standard pretesting showed that adding a cognitive bolt-on to the EQ-5D-Y increased the explanatory power for the entire group and for those with RD and T1D separately. Therefore, the addition of the bolt-on is beneficial across both disease groups. The bolt-on items have a higher discriminatory power than the standard items of the EQ-5D-Y. However, internal validity analysis showed that omitting the memory item leads to improved internal consistency. This result suggests that cognition may be a multi-dimensional concept and the item “memory” may measure content that the other three items are not able to capture. Similarly, the factor analysis suggests that the four items of the bolt-on are based on one or two latent dimensions. The results of both the internal validity and factor analysis support that the bolt-on items could be reduced to two dimensions. Furthermore, regression analyses showed that the combination of “concentration” and “memory” yields the greatest increased explanatory power for any two items. This combination is further supported by the NGT results of the focus groups where these cognitive components were weighted the highest.

The results of the regression analysis could further support reducing the bolt-on items as the coefficients for problems with items of “concentration” and “memory” counter-intuitively increased the EQ-VAS, or improved general health. As the bolt-on items show strong linear correlations with each other, the estimated coefficients in the linear regression model in this study may suffer from multicollinearity, which can lead to reversed signs in the coefficients. Further research could explore whether reducing the items leads to more intuitive effects.

Based on the literature reviews and the empirical results of this study, one could argue for the inclusion of the EQ-5D-Y cognitive bolt-on in people living with T1D and RD. This could improve the HRQoL measurement in children and adolescents with these conditions. As literature suggests that children with acute and other chronic illness may have cognitive difficulties, it should be empirically tested which other health conditions may also benefit from the inclusion of the developed cognitive bolt-on in HRQoL measurement. However, a key feature of the EQ-5D instruments is that it can be used throughout childhood and adolescence until adulthood. Furthermore, the generic approach of the instrument enables comparisons between different health conditions and its application in economic evaluation for health-decision making. The disadvantage of adding a bolt-on to the EQ-5D-Y for certain health conditions is a reduction of this comparability to EQ-5D-Y outcomes in other disease areas.

In addition to the aforementioned limitation regarding the inclusion of mainly children and adolescents with T1D or RD, the selection of the most frequently used instruments was based on existing reviews and a manual literature search which may have influenced the consideration for cognitive items and components thereof. It is possible that other child-friendly HRQoL instruments including a cognitive dimension were not considered. The development of the bolt-on was completed in 2010 and the CHU-9D has since been developed and is currently one of the most frequently used HRQoL instruments [39]. Considering the content of the CHU-9D cognitive dimension it includes one item of school performance (setting school) [40, 41] and thus would not have altered the literature review results. Furthermore, the development and item selection of HRQoL instruments is mostly non-transparent and as such the review of instruments had a smaller contribution to the development of the bolt-on. Instead, development was based mainly on the empirical results of this study. The completion of the second questionnaire in phase 2 of the pretest was done at home and may not have been completed on day five (even though the participants received a reminder call on day five). Furthermore, participants’ health status may have changed between the completion of the two measures.

The study was conducted solely in Germany and the results cannot be generalized. Further research is needed to confirm these results in other countries. The questionnaire should be translated and tested in other (multi-)national studies. Although the study results yielded good acceptability, feasibility and validity, additional research investigating the psychometric properties of the extended instrument in other disease areas, an inpatient and general population sample of children and adolescents is needed. Furthermore, test–retest analysis nor responsiveness was investigated for the EQ-5D-Y plus bolt-on or compared to the EQ-5D-Y and should be objectives of future studies.

## Conclusions

By enhancing the EQ-5D-Y with the cognitive bolt-on, we developed a measurement instrument that incorporates current research results on HRQoL and is suitable for the target population. The empirical results of this study show that cognitive functioning is an important part of HRQoL assessment in children and adolescents. Given that an appropriate measuring instrument should represent the HRQoL dimensions relevant to the target group, the inclusion of a cognitive dimension in the EQ-5D-Y improves the HRQoL measurement.

For paediatric patients living with RD or T1D, the EQ-5D-Y plus bolt-on demonstrated good acceptability, feasibility and validity. The cognitive bolt-on items improved the explanatory power of the EQ-VAS. Factor analysis showed that a reduction of the bolt-on to one or two items is justifiable. Future research should further investigate the selection of the bolt-on items for the EQ-5D-Y and the transferability of results.

## Supplementary Information

Below is the link to the electronic supplementary material.

Supplementary file1 (DOCX 110 kb)

## Data Availability

Data collected in this study are available from the corresponding author upon reasonable request.

## Code availability

Code generated in this study is available from the corresponding author upon reasonable request.
