# Supplementary material for: Developing and testing a cognitive bolt-on for the EQ-5D-Y (Youth)
Source: Qual Life Res. 2021 Jun 10;31(1):215–29. doi: 10.1007/s11136-021-02899-x (PMC8800913; doi:10.1007/s11136-021-02899-x)
Supplement: Supplementary file 1 — Supplementary file1 (DOCX 110 kb) [file 11136_2021_2899_MOESM1_ESM.docx]

**Appendix: Development and testing a cognitive bolt-on for the EQ-5D-Y**

The development and testing of the EQ-5D-Y plus cognitive bolt-on was conducted across several methodological steps (see Figure A1).

*Figure A1: Overview of the methodological procedure*


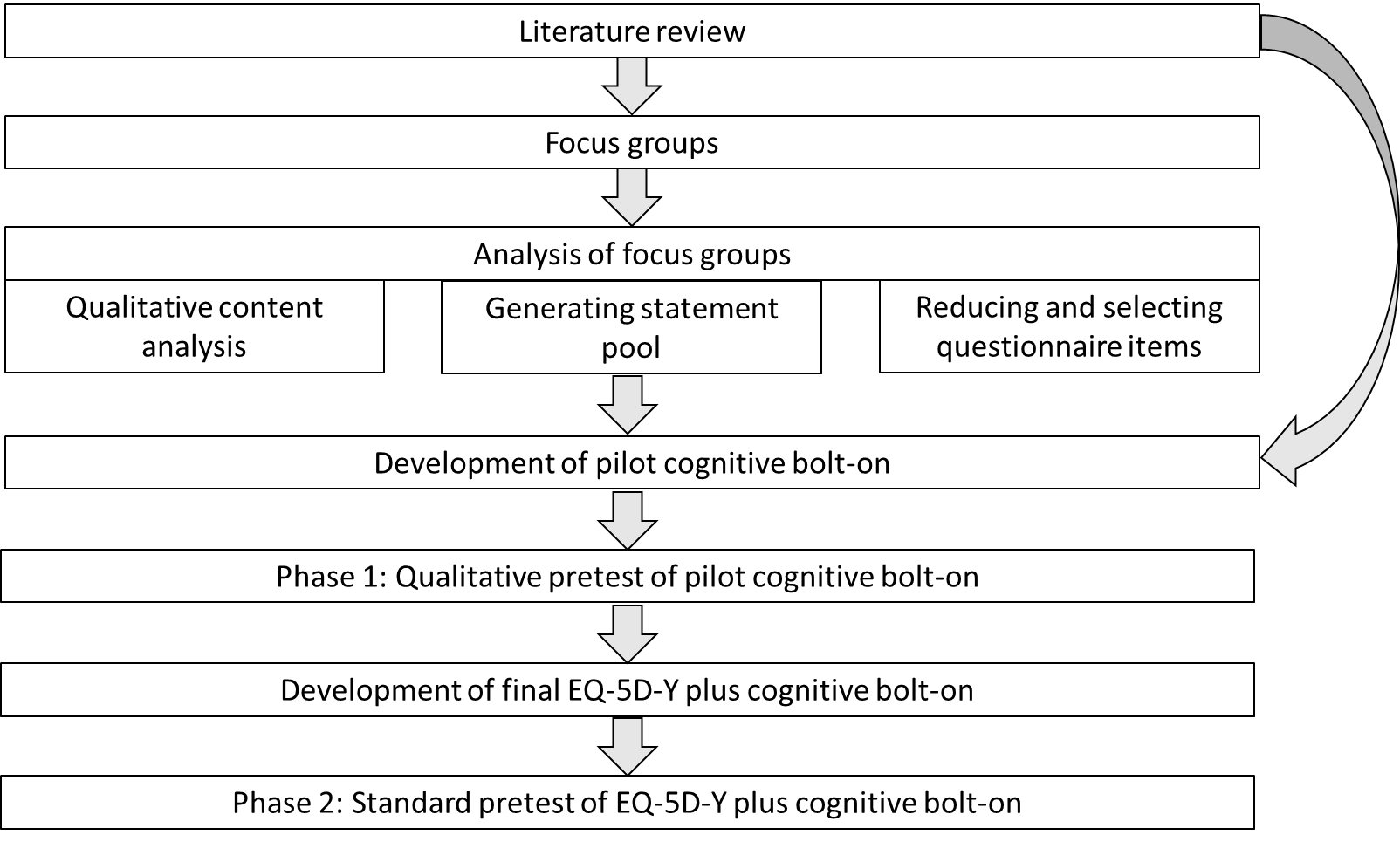


[Figure A1 here]

**Development of the cognitive bolt-on**

***Literature reviews***

Two sequential non-systematic literature reviews were conducted to identify, (1) components of cognitive functioning, and (2) items and dimensions encompassing cognitive functioning included in existing HRQoL instruments for children and adolescents. The results of the first non-systematic literature review which identified components of cognitive functioning, in psychology research, were discussed with three paediatric psychologists. In the second non-systematic literature review, the selection of instruments was informed by two existing reviews of HRQoL instruments [1, 2] and a manual literature search. Only self-report versions of published instruments which (a) were specifically designed for children and adolescents and (b) contain a cognitive dimension or item were included. Seven instruments met the inclusion criteria (Child Health Questionnaire (CHQ) [3]; Child Health and Illness Profile (CHIP) [4]; How are you? (HAY) [5], KIDSCREEN [6, 7]; KINDL-R [8]; Pediatric Quality of Life Inventory (PedsQL) [9, 10] and TNO/AZL Child Quality of Life (TACQOL) [11]). Analysis focused on number, content and wording of cognitive items. The results from the literature reviews were included in the topic guide for the focus groups.

***Focus groups***

Focus groups with children and adolescents were conducted by the research team to investigate the understanding and importance of the components of cognitive functioning, and to identify items for the bolt-on. A double-layer design was used: 2 x 3 focus groups (layer one: aged 8 to 11 and 12 to 15; layer two: with/without special educational needs and chronic conditions which may impact cognitive abilities). It was assumed that children with impairments would be more open about their experiences if grouped with other children living with the same condition. Furthermore, more active participation was expected from younger children if grouped with others their age. Participants were recruited from three schools and one hospital in Germany.

Each focus group included discussion around 1) their daily activities; 2) the impact of (their) health condition on daily activities; 3) components of cognitive functioning and finally 4) the components of cognitive functioning were weighted using nominal group technique (NGT) (i.e. selection of three most relevant components) [12, 13]. Participants were asked to imagine they were “mixing a fitness drink” that would help them work at a high cognitive level. They were asked to select the three most relevant “ingredients”, or components of cognitive functioning, for that purpose. The most relevant component was valued with 3 points, the second with 2 points and the third one with 1 point.

The focus groups were recorded and transcribed, then analysed using qualitative content analysis according to Mayring (steps of (a) paraphrasing, (b) generalising, and (c) reducing with deductive category application) [14]. A pool of possible items for the bolt-on was derived from the participants’ statements (i.e. candidate sub-domains without specific terminology and framing). Items were reduced and selected by the research team using the card sorting procedure [15]. Statements were grouped together based on shared characteristics and rephrased into items appropriate for questionnaire completion. Thereafter, items were reduced by applying reduction criteria in two steps: (1) *redundancy* and *concept of cognitive functioning* and (2) *applicability, relevance and clarity*. The analysis of the focus groups was stratified by age group to investigate the need for different age versions.

***Development of cognitive dimension***

The components of cognitive functioning and the respective items were selected for the pilot bolt-on with consideration of the results from both literature reviews, the qualitative analysis of the focus groups and the NGT (cumulative weighting of components of cognition across focus groups). The development of items further considered relevance to the target group by incorporating components of cognition with an explanation taken from the children and adolescent’s explanation/understanding of the items. The wording, structure and design of the bolt-on items followed the EQ-5D-Y.

***Pretesting the bolt-on items***

Phase 1

In phase 1 of the pretest, the selected bolt-on items were formatted in the EQ-5D style (i.e. wording of items, explanation in parentheses and response level) and then assessed for acceptability and feasibility (in terms of comprehensibility). Qualitative face-to-face interviews using cognitive techniques ((a) read aloud and paraphrasing, (b) general probing, and (c) category selection probing) were conducted [16]. These interviews used the same double-layer-design, age range and recruiting method as the focus groups.

The interviews consisted of two parts: completion of the EQ-5D-Y plus bolt-on and semi-structured individual interviews, in which the participants were asked to judge the importance or redundancy of the cognitive items. The participants’ responses and questions of clarification during the completion of the questionnaire were noted. The semi-structured interviews were recorded and transcribed for content analysis according to Mayring (steps of (a) paraphrasing, (b) generalising and (c) reducing with deductive category application) [14]. Descriptive analyses were used to examine the frequencies of reported problems for the bolt-on items. Statistical analyses were performed in R. Based on the results of the qualitative pretest, the bolt-on was adapted and finalised for further testing.

***Phase 2: Standard pretest***

In the second pretest phase, a quantitative survey was conducted in a pediatric outpatient clinic in Germany to test the acceptability, feasibility, and performance of the newly developed EQ-5D-Y plus bolt-on. Children aged 8 to 15 living with type 1 diabetes mellitus (T1D) or a rheumatic disorder (RD) were enrolled. The selection of disease groups was according to prevalence in children and adolescents and for the associated impact on cognitive function.

Each participant self-completed both EQ-5D-Y and EQ-5D-Y plus bolt-on. The questionnaires were completed at different time points: day 1 (after a medical check-up) and day 5 (at home). Participants’ HRQoL was additionally evaluated with the KIDSCREEN-27 [17]. The order of completion was randomised to reduce bias.

This phase explored the following research questions:

(1) Does the addition of a cognitive bolt-on improve the explanatory power of the questionnaire compared to the standard EQ-5D-Y?

(2) Does the benefit of measuring HRQoL with an additional cognitive bolt-on differ between children and adolescents living with RD and T1D?

Descriptive analyses (proportions for discrete variables, mean and standard deviation (SD) for continuous variables) were used to examine the sample characteristics and the responses to the EQ-5D-Y and EQ-5D-Y plus bolt-on. The feasibility and acceptability of EQ-5D-Y plus bolt-on was investigated by missing values in terms of non-response and ceiling effects. Ceiling effects were defined as the proportion of reported "no problems" excluding missing values. Differences between questionnaire versions and the disease groups were identified via t-test or Mann-Whitney-U-test.

The explanatory power or contribution of the bolt-on items, in addition to the five standard items, to the self-rated overall health status measured by the EQ-VAS was explored through linear regression. To compare the explanatory power of the EQ-5D-Y to the EQ-5D-Y plus bolt-on, the following linear regression models were calculated:

1. $Y_{i}= \beta_{0}+\beta_{1}X_{1i}+\beta_{3}{age}_{i}+\beta_{4}{diag}_{i}+\varepsilon_{i}$
2. $Y_{i}= \beta_{0}+\beta_{1}X_{1i}+\beta_{2}X_{2i}+\beta_{3}{age}_{i}+\beta_{4}{diag}_{i}+\varepsilon_{i}$

where $Y_{i}$ corresponds to the value on the EQ-VAS of respondent$i$, with$i=1, \ldots, n$. The vector $X_{1}$ corresponds to the vector of dummy variables of the five EQ-5D-Y items and $X_{2}$ corresponds to the dummy variables of the bolt-on items. The variable ${age}_{i}$ measures the age of respondent $i$ and ${diag}_{i}$ is a dummy variable that indicates whether individual $i$ is living with either RD or T1D. The comparison of explanatory power of the two models was achieved by comparing their coefficients of determination ($R^{2}$,$R_{adj}^{2}$) and Akaike’s information criterion (AIC).

The Shannon Index (H') and the Shannon Evenness Index (J') were used to evaluate the discriminatory power of the bolt-on in terms of absolute and relative informativity [18, 19]. The indices were calculated for (1) the EQ-5D-Y plus bolt-on, (2) the EQ-5D-Y and (3) the bolt-on, as well as (4) for each of the nine items of the EQ-5D-Y plus bolt-on. In order to assess the external validity of the bolt-on, Spearman rank correlations were calculated between the bolt-on items and the cognitive items from the validated KIDSCREEN-27 [20]. Internal consistency of the bolt-on items was measured by Cronbach’s α.

Factor analysis was used to test whether the items of the bolt-on could be reduced to one or two latent factors. Using various rotation methods, factor loadings of the individual items or combinations of items were determined for different number of factors. Finally, linear regression models were fitted, in which the factors resulting from the factor analysis were included as regressors. $R^{2}$,$R_{adj}^{2}$ and AIC of the respective models were compared to determine the items that yield the best model fit and added the most explanatory power to the model.

All statistical analyses were performed in R version 3.6.1 [21]. The level of significance was set at p<0.05.

**References**

1. Ravens-Sieberer, U., Erhart, M., Wille, N., Nickel, J., & Bullinger, M. (2007). Lebensqualitätsverfahren für Kinder – methodische Herausforderungen und aktuelle Instrumente: Quality of Life Measures for Children – Methodological Challenges and State of the Art. *Zeitschrift für Medizinische Psychologie,* 16(1,2), 25–40.

2. Rajmil, L., Herdman, M., Fernandez de Sanmamed, M.-J., Detmar, S., Bruil, J., Ravens-Sieberer, U., et al. (2004). Generic health-related quality of life instruments in children and adolescents: a qualitative analysis of content. *Journal of Adolescent Health,* 34(1), 37–45. doi:10.1016/S1054-139X(03)00249-0.

3. Landgraf, J. M., Abetz, L., & Ware, J. E. (1996). *The CHQ User’s Manual* (1st edn). Boston: The Health Institute, New England Medical Center.

4. Riley, A. W., Forrest, C. B., Rebok, G. W., Starfield, B., Green, B. F., Robertson, J. A., et al. (2004). The Child Report Form of the CHIP-Child Edition: reliability and validity. *Medical care,* 42(3), 221–231. doi:10.1097/01.mlr.0000114910.46921.73.

5. Bruil, J. (1999). *Development of a quality of life instrument for children with a chronic illness*. Zugl.: Leiden, Univ., Diss., 1999 (Health psychology series, Vol. 7). Leiden: Univ.

6. Ravens-Sieberer, U., Auquier, P., Erhart, M., Gosch, A., Rajmil, L., Bruil, J., et al. (2007). The KIDSCREEN-27 quality of life measure for children and adolescents: psychometric results from a cross-cultural survey in 13 European countries. *Quality of life research,* 16(8), 1347–1356. doi:10.1007/s11136-007-9240-2.

7. Ravens-Sieberer, U., Gosch, A., Rajmil, L., Erhart, M., Bruil, J., Power, M., et al. (2008). The KIDSCREEN-52 quality of life measure for children and adolescents: psychometric results from a cross-cultural survey in 13 European countries. *Value in health,* 11(4), 645–658. doi:10.1111/j.1524-4733.2007.00291.x.

8. Ravens-Sieberer, U., & Bullinger, M. (1998). Assessing health-related quality of life in chronically ill children with the German KINDL: first psychometric and content analytical results. *Quality of life research,* 7(5), 399–407. doi:10.1023/a:1008853819715.

9. Varni, J. W., Seid, M., & Rode, C. A. (1999). The PedsQL: measurement model for the pediatric quality of life inventory. *Medical care,* 37(2), 126–139. doi:10.1097/00005650-199902000-00003.

10. Varni, J. W., Seid, M., & Kurtin, P. S. (2001). PedsQL 4.0: reliability and validity of the Pediatric Quality of Life Inventory version 4.0 generic core scales in healthy and patient populations. *Medical care,* 39(8), 800–812. doi:10.1097/00005650-200108000-00006.

11. Vogels, T., Verrips, G. H., Verloove-Vanhorick, S. P., Fekkes, M., Kamphuis, R. P., Koopman, H. M., et al. (1998). Measuring health-related quality of life in children: the development of the TACQOL parent form. *Quality of life research,* 7(5), 457–465. doi:10.1023/a:1008848218806.

12. van den Bemt, L., Kooijman, S., Linssen, V., Lucassen, P., Muris, J., Slabbers, G., et al. (2010). How does asthma influence the daily life of children? Results of focus group interviews. *Health and quality of life outcomes,* 8, 5. doi:10.1186/1477-7525-8-5.

13. Ronen, G. M., Rosenbaum, P., Law, M., & Streiner, D. L. (2001). Health-related quality of life in childhood disorders: a modified focus group technique to involve children. *Quality of life research,* 10(1), 71–79. doi:10.1023/a:1016659917227.

14. Mayring, P. (2015). *Qualitative Inhaltsanalyse: Grundlagen und Techniken* (12th edn, Beltz Pädagogik). Weinheim: Beltz.

15. Canter, D., & Brown, J. & Groat, L. (1985). A Multiple Sorting Procedure for Studying Conceptual Systems. In M. Brenner & Brown, J. & Canter, D. (Eds.), *The Research Interview: Uses and Approaches*. London: Academic Press.

16. Prüfer, Peter & Rexroth, Margrit (2000). *Zwei-Phasen-Pretesting: (ZUMA-Arbeitsbericht, 2000/08)*. Mannheim.

17. Ravens-Sieberer, U. (2016). *The Kidscreen questionnaires: Quality of life questionnaires for children and adolescents: handbook* (3rd edn). Lengerich: Pabst Science Publishers.

18. Shannon, C. E. (1948). A Mathematical Theory of Communication. *Bell System Technical Journal,* 27(3), 379–423. doi:10.1002/j.1538-7305.1948.tb01338.x.

19. Janssen B., M. F., Birnie, E., & Bonsel, G. J. (2007). Evaluating the discriminatory power of EQ-5D, HUI2 and HUI3 in a US general population survey using Shannon's indices. *Quality of life research,* 16(5), 895–904. doi:10.1007/s11136-006-9160-6.

20. Ravens-Sieberer, U., Wille, N., Badia, X., Bonsel, G., Burström, K., Cavrini, G., et al. (2010). Feasibility, reliability, and validity of the EQ-5D-Y: results from a multinational study. *Quality of life research,* 19(6), 887–897. doi:10.1007/s11136-010-9649-x.

21. R Core Team (2019). *R: A language and environment for statistical computing*. Vienna, Austria: R Foundation for Statistical Computing.
